# Early Impact of a National Multi-Faceted Road Safety Intervention Program in Mexico: Results of a Time-Series Analysis

**DOI:** 10.1371/journal.pone.0087482

**Published:** 2014-01-31

**Authors:** Aruna Chandran, Ricardo Pérez-Núñez, Abdulgafoor M. Bachani, Martha Híjar, Aarón Salinas-Rodríguez, Adnan A. Hyder

**Affiliations:** 1 International Injury Research Unit, Johns Hopkins Bloomberg School of Public Health, Baltimore, Maryland, United States of America; 2 Centro de Investigación en Sistemas de Salud, Instituto Nacional de Salud Pública, Cuernavaca, Morelos México; 3 Fundación Entornos A.C., Cuernavaca, Morelos, México; 4 Centro de Investigación en Evaluación y Encuestas, Instituto Nacional de Salud Pública, Cuernavaca, Morelos México; University of California, San Francisco, United States of America

## Abstract

**Background:**

In January 2008, a national multifaceted road safety intervention program (IMESEVI) funded by the Bloomberg Philanthropies was launched in Mexico. Two years later in 2010, IMESEVI was refocused as part of a 10-country international consortium demonstration project (IMESEVI/RS10). We evaluate the initial effects of each phase of the road safety intervention project on numbers of RT crashes, injuries and deaths in Mexico and in the two main target cities of Guadalajara-Zapopan and León.

**Methods:**

An interrupted time series analysis using autoregressive integrated moving average (ARIMA) modeling was performed using monthly data of rates of RT crashes and injuries (police data), as well as deaths (mortality system data) from 1999–2011 with dummy variables representing each intervention phase.

**Results:**

In the period following the first intervention phase at the country level and in the city of León, the rate of RT crashes decreased significantly (p<0.05). Notably, following the second intervention phase although there was no reduction at the country level, there has been a decrease in the RT crash rate in both Guadalajara-Zapopan (p = 0.029) and in León (p = 0.029). There were no significant differences in the RT injury or death rates following either intervention phase in either city.

**Conclusion:**

These initial results suggest that a multi-faceted road safety intervention program appears to be effective in reducing road crashes in a middle-income country setting. Further analysis is needed to differentiate the effects of various interventions, and to determine what other economic and political factors might have affected this change.

## Introduction

Road traffic (RT) crashes result in a significant morbidity and mortality burden worldwide; according to the the most recent Global Disease Burden estimates, the global age-adjusted mortality rate from road traffic injuries was 19.5 per 100 000 [1]. Mexico has among the highest RT crash burden in the Americas region; in 2010, Mexico’s adjusted RT mortality rate was 17.2 per 100 000 population, and the annual road traffic crash rate was 394 per 100 000 population [2], [3]. According to the Mexican 2012 National Nutrition and Health Survey, the reported prevalence of non-fatal RT injuries was 1.2% (95% CI: 1.1–1.3); this figure of 1.4 million Mexicans having been injured in a RT crash in the year prior to the survey is higher than what has been reported previously [4], [5].

Recognizing the need to address this important public health problem, in July 2008, the WHO partnered with the Mexican Ministry of Health and the National Center for Accident Prevention (CENAPRA) to launch the *Iniciativa Mexicana de Seguridad Vial y Prevención de Lesiones en el Tránsito* (IMESEVI) funded by the Bloomberg Philanthropies (New York, NY). Prior road safety intervention efforts in Mexico focused on a variety of state and local level efforts that resulted in varying degrees of success [6]–[11]. IMESEVI utilized an approach of combining several evidenced-based policies into a comprehensive multi-pronged effort; similar “packaged” approaches have been used in other settings; for example, in Spain an effort launched in 2004 resulted in a reduction in road traffic injuries in all non-pedestrian road users [12]–[14].

The goal of the IMESEVI project was to reduce the burden of injuries and deaths from road traffic crashes. The program focused in four cities, two of which were Guadalajara (Jalisco) and León (Guanajuato). Interventions involved augmenting enforcement of drink-driving as well as campaigns to promote seatbelt and child restraint use. In January 2010, the Bloomberg Philanthropies funded a consortium of partners with the aim of improving road safety across 10 countries; Mexico was included as one of the 10 countries [15]. This second phase of the IMESEVI (RS-10; now known as the Global Road Safety Program) project was launched in January 2010, and once again, two of the six cities of focus were chosen to be the metropolitan area of Guadalajara-Zapopan and León. In this phase in these two cities, drink-driving enforcement and legislation were the focus of the first year, followed by the addition of seatbelt and child restraint campaigns in the second year.

Time series analysis has been shown to be an optimal method for interpreting changes in trends associated with community-based interventions in many settings [16], [17]. A recent analysis from Brazil utilized this method to analyze the effects of lowering the legal blood alcohol limit on RT injuries and deaths [18]; an interrupted time series analysis allowed interpretation of changes in trends associated with the intervention while accounting for other influencing factors given the less-than-ideal data available for analysis.

The aim of this investigation was to assess the early effectiveness of the two phases of Mexico’s IMESEVI initiative on RT crashes and deaths at the country level in Mexico and in two of its target municipalities of Guadalajara-Zapopan and León. To our knowledge, this represents one of the first analyses of the impact of a widespread community based road safety intervention in Mexico.

## Methods

### Study Design and Setting

**Figure 1:**
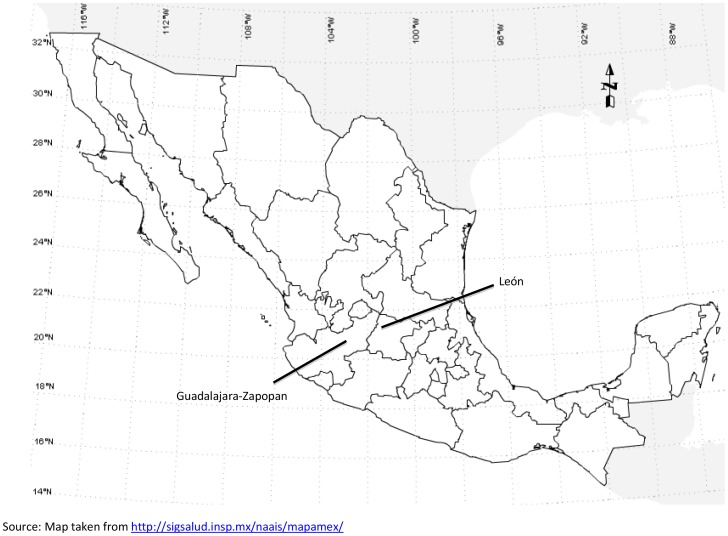
Map of Mexico showing the target intervention areas of Guadalajara-Zapopan and León.

This is a plausibility evaluation of the early impact of the first and second phases of the IMESEVI program. Both phases of the program focused primarily on drink-driving enforcement and promotion of seatbelts and child restraint use in the two target cities. In addition, the 2^nd^ phase of IMESEVI also included a youth-focused intervention program in which youth were mobilized to serve as peer educators and resources for the promotion of road safety. Guadalajara is the capital of the state of Jalisco in Mexico’s western-pacific; it is the second most populous Mexican city. The Guadalajara metropolitan area has extended beyond its borders and absorbed many formerly outlying communities, including Zapopan. In 2010, the population of Guadalajara-Zapopan was 2835 953, and the 2011 fleet size was 1.4 million registered motor vehicles. León, located in the center of Mexico, is the 7th most populous city and the most populous in the state of Guanajuato although it is not the state’s capital. In 2010, León’s population was 1426 865, and the 2011 fleet size was over 379 000 registered motor vehicles (Figure 1).

### Variables and Data Sources

Traffic crash and injury data were obtained from the database of road traffic crashes in urban and suburban zones (ATUS) reported by the Mexican National Institute of Geography and Statistics (INEGI) [2]. This database of crash statistics includes all road traffic crashes that are registered by the municipal authorities from the transport and police sectors. Forms completed at the crash site are entered into a regional database, and then sent to the national level. The vast majority of municipalities report to INEGI. Mortality data is aggregated, validated, and reported also by INEGI from death certificates filed by the Ministry of Health. Information that had been validated by INEGI was analyzed in this study. Data was extracted by month from January 1999– December 2011 as reported at the national level and for each of municipalities of Guadalajara, Zapopan, and León. National and local population estimates were obtained from the National Health Information System (SINAIS) from the Ministry of Health [19]. Monthly road traffic crash, injury and death rates were constructed using mid-year population estimates of each year.

We used a dummy variable to represent the different phases on the interventions: “0” for the observation period before IMESEVI started (January 1999–February 2008), “1” representing the initiation of the first phase of IMESEVI (March 2008–December 2009) and “2” for the second phase of IMESEVI/RS-10 (January 2010–December 2011). In addition, we included the motorization rate per 1 000 inhabitants, which was obtained from official registered fleet size records, compiled by INEGI [20]. To adjust for alcohol consumption, we used information on the special tax for the production and distribution of alcoholic beverages for the period under study (IEPS), obtained from the *Secretaría de Hacienda y Crédito Público* (Secretariat of Finance and Public Credit). Of note, IEPS information is only available at the national level and it is aggregated by year. IEPS figures were translated to December 2012 peso rates using the Consumer Price Index reported by *Banco de México* (http://www.banxico.org.mx) and then divided by the population estimate of each year.

### Statistical Analysis

This longitudinal study used as control groups both pre-intervention figures of the two cities under study and national figures [21]. An interrupted time series analysis was performed using fitted autoregressive integrated moving average (ARIMA) models with robust standard errors for Mexico and for the two cities of interest. An annual difference operator (S12) was used to account for seasonal variation [22]. The Dickey-Fuller test was used to confirm if the data was stationary [23].

Initial auto-regressive (AR) and moving-average (MA) structures were chosen based on examining the autocorrelation (AC) and partial autocorrelation (PAC) of time series. Terms were then removed sequentially based on the statistical significance and the models were re-run. The final model was chosen using the Akaike Information Criterion (AIC). The residuals of the final model were tested using both, the Bartlett’s and Pormendiou’s white noise Q tests and also evaluated by examining the autocorrelation (AC) and partial autocorrelation (PAC) of estimated residuals. All models were fitted using Stata Version 12.1 (StataCorp, L.P.), and a p<0.05 was considered significant.

## Results

The location of the target cities within Mexico is shown in Figure 1. More than 5.3 million collisions were reported in Mexico from 1999 to 2011; 464 631 (9%) of them occurred in Guadalajara-Zapopan and 107 188 (2%) in León. Almost 1.8 million people were injured as a result; 41 138 in Guadalajara-Zapopan and 32 923 in León. A total of 203 053 people died due to a RT crash in the country; 7 271 (3.6%) in Guadalajara-Zapopan and 2 535 (1.2%) in León.

**Figure 2:**
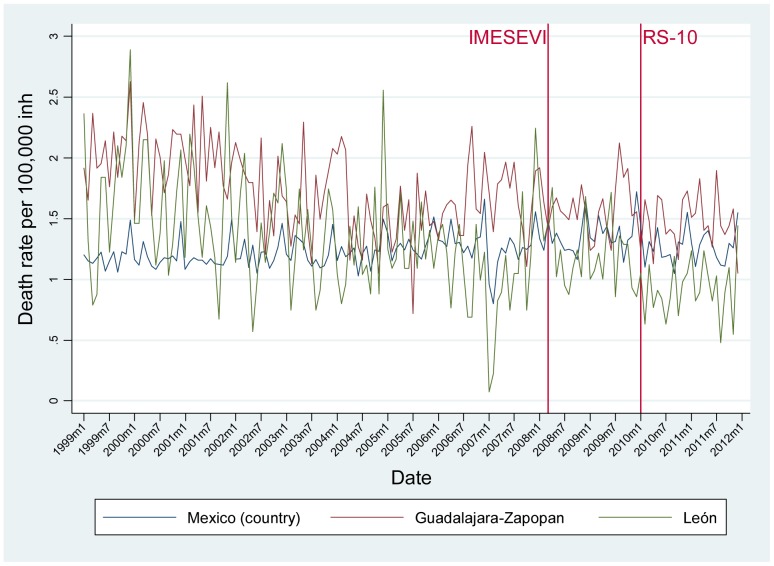
Time trend of mortality rates by month in Mexico (country), León and Guadalajara-Zapopan before and after phases of a road safety intervention program, 1999–2011.

**Figure 3:**
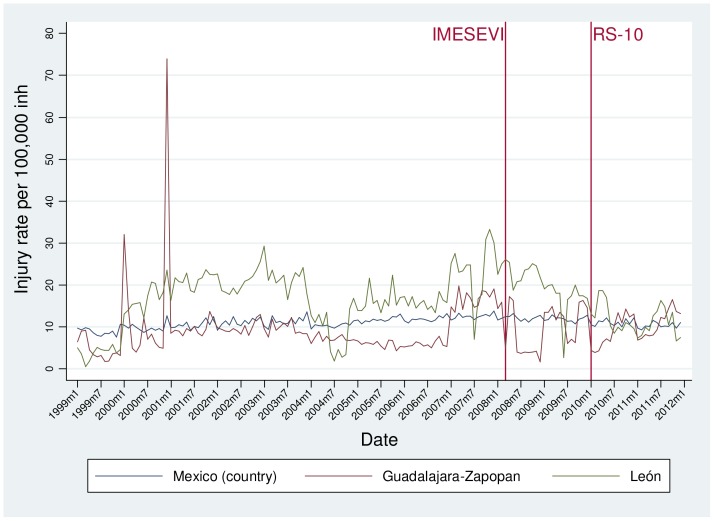
Time trend of injury rates by month in Mexico (country), León and Guadalajara-Zapopan before and after phases of a road safety intervention program, 1999–2011.

**Figure 4:**
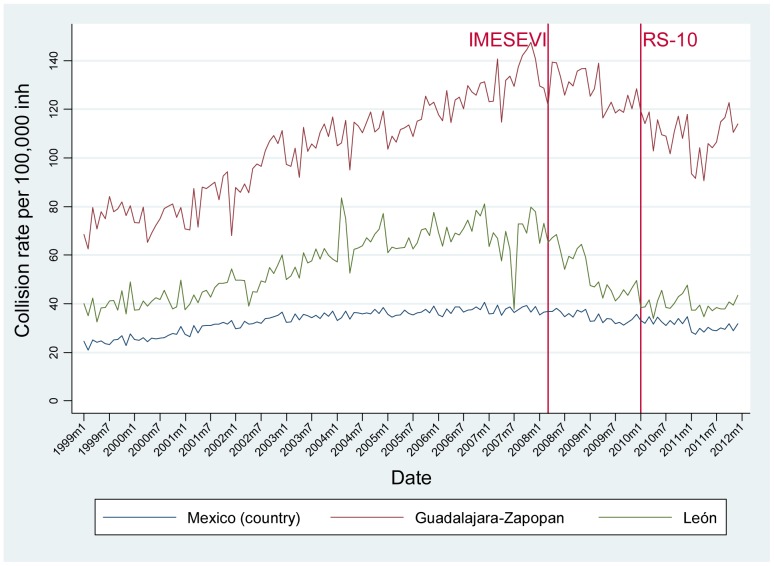
Time trend of collision rates by month in Mexico (country), León and Guadalajara-Zapopan before and after phases of a road safety intervention program, 1999–2011.

The monthly RT death, injury and crash rates for each municipality as well as at a national level are shown in Figures 2, 3 and 4, respectively. Between 1999 and 2011, the RT mortality rate (Figure 2) has remained stable for the three areas, although some seasonal trends are seen with increases in the summer months. This is also the case for the injury rate in Mexico (Figure 3). In León, the injury rate decreased from January 2003 (29.29 per 100,000) to July 2004 (1.92 per 100,000), and then peaked again in November 2007 (33.28 per 100,000), after which the trend shows an apparent decrease. In Guadalajara-Zapopan, some outlier values are observed compared to the overall mean of 9.52 per 100 000; in January 2000 the RT injury rate was 32.04 per 100,000 and in December 2000 it was 73.87 per 100 000. Finally, it is evident that the RT crash rate steadily increased in the three settings from 1999–2008, and then decreased for the subsequent two years (Figure 4). The declines have not continued after 2011 in Guadalajara-Zapopan.

The time series analysis showed no significant differences in the RT mortality rate in the country, León or Guadalajara-Zapopan following the initiation of the 1^st^ or 2^nd^ intervention phases. The RT injury rate also remained the same after both phases of interventions in the two cities of interest, although in the country the RT injury rate appeared to be lower after the second intervention phase. In terms of RT crashes, the time series analysis showed that in the period following the first intervention phase in Mexico and León, the rate of RT crashes decreased significantly (p<0.05) (Table 1). Although there was no significant change in RT crashes after the second phase of intervention at the country level, both Guadalajara-Zapopan and León showed a significant decrease.

**Table 1:** Interrupted autoregressive integrated moving average (ARIMA) modeling to estimate effects of IMESEVI and RS-10 interventions in Mexico and two targeted cities.

	Estimate	SE	p-value
**Death rate**			
*** México (country) Model: AR(1) S(12) MA (1 2 12)***			
IMESEVI	0.03	0.04	0.546
IMESEVI/RS-10	−0.06	0.04	0.114
Motorization rate	0.00	0.00	0.819
Annual *IEPS*	199.50	453.52	0.660
*** Guadalajara-Zapopan Model: AR(4) S(12) MA (12)***			
IMESEVI	−0.04	0.08	0.576
IMESEVI/RS-10	−0.12	0.08	0.125
Motorization rate	0.00	0.00	0.013*
*** León Model: AR(1 4 12) S(12) MA (4 12)***			
IMESEVI	0.00	0.12	0.977
IMESEVI/RS-10	−0.09	0.13	0.474
Motorization rate	0.00	0.00	0.412
**Injury rate**			
*** México (country) Model: AR(4 12) S(12) MA (1/5)***			
IMESEVI	−0.49	0.38	0.193
IMESEVI/RS-10	−1.21	0.41	0.003*
Motorization rate	0.00	0.01	0.538
Annual *IEPS*	−8844.09	5576.83	0.113
*** Guadalajara-Zapopan Model: AR(11 12) S(12) MA (11 12)***			
IMESEVI	−2.21	1.98	0.264
IMESEVI/RS-10	−1.92	1.56	0.219
Motorization rate	0.01	0.01	0.156
*** León Model: AR(12) S(12) MA (1/5)***			
IMESEVI	0.07	3.72	0.985
IMESEVI/RS-10	−0.32	3.99	0.937
Motorization rate	−0.07	0.05	0.112
**Collision rate**			
*** México (country) Model: AR(3 12) S(12) MA (1/7 9)***			
IMESEVI	−0.90	0.40	0.026*
IMESEVI/RS-10	−0.26	0.71	0.718
Motorization rate	−0.03	0.01	0.000*
Annual *IEPS*	−2906.24	10696.18	0.786
*** Guadalajara-Zapopan Model: AR(1 2 12) S(12) MA (1/7 10 11)***			
IMESEVI	−5.29	3.80	0.164
IMESEVI/RS-10	−10.39	4.75	0.029*
Motorization rate	−0.03	0.02	0.106
*** León Model: AR(12) S(12) MA (1 3 4 6)***			
IMESEVI	−12.21	3.11	0.000*
IMESEVI/RS-10	−5.73	2.63	0.029*
Motorization rate	−0.04	0.03	0.190

SE: standard error; AR: autocorrelation with (#*p*) lags of autocorrelations; S: Seasonality; MA: Moving Average with (#*q*) lags of moving averages; IEPS: National alcohol production and distribution. *Denotes those values that are statistically significant at p<0.05.

## Discussion

In comparing RT crashes, injuries and deaths at a country level and in two of the target intervention cities following two phases of a multi-faceted road safety intervention program in Mexico, our results show a decrease in RT crashes in the target cities in the initial two years of the 2^nd^ phase (IMSEVI/RS10) of the intervention. The overall crash rate for the entire country as well as for the municipality of León did decrease following the 1^st^ intervention phase; it is important to note many aspects of IMESEVI have not been national in scope. Of note, this decrease did not continue at the national level after the 2^nd^ intervention phase, but there was a decrease in both Guadalajara-Zapopan and León. Despite what one might assume would be a correlation between reduced RT crashes and numbers of RT deaths, we did not see a change in the RT injury or death rates following either phase of the intervention in either municipality thus far; it could be that more time will be needed to observe such changes.

Two of the major risk factors of focus during both phases of the IMESEVI project were drinking and driving and seatbelt/child restraint use. Alcohol-associated driving has been shown to be associated with both RT crash likelihood and severity [24], and seatbelts have been shown to reduce RT deaths [25]. Some changes did occur in the legislation and enforcement of alcohol-associated driving in these municipalities during the time period of this analysis and afterwards; in September 2010 the state of Jalisco passed a law lowering the legal blood alcohol limit for drivers [26] which was then substituted by the new legislation in August 2013 [27]. It would be interesting to know the differential effects of specific interventions related to these and other risk factors on the RT crash rate, as well as the influence of relevant changes in risk factor behavior due to other factors outside of the specific intervention. This information would be important as new community based interventions are planned in alternate low and middle income country settings.

Our study has several limitations. First, we did not include outside influencing variables such as annual income; the global recession that occurred in 2008 might have influenced overall driving behavior thereby affecting the RT crash rate. However, the registered motor vehicle fleet size continued to increase after 2008 (going from 29.3 million in 2008 to 33.2 million in 2011 at the country-level). Therefore, one might predict that driving patterns may not have been significantly affected by the economic downturn. Second, our ability to assess changes in important risk factor behaviors during this time such as alcohol consumption was limited. There was no information regarding alcohol consumption available at the local/state level. Although we attempted to include a proxy variable accounting for alcohol consumption at the national level, this information was aggregated by year and thus we were not able to account for seasonal variations in this indicator. Further analyses are needed to determine what other economic or political factors might have affected this change. Third, we were limited by the quality of secondary information that is available in Mexico. It has been shown that both RT injury and mortality figures in Mexico likely significantly underestimate the true burdens [3], [28]. However, this is the best available systematically collected information in the country; ongoing efforts to improve the quality of this data are essential.

Time series ARIMA models have been shown to be useful and appropriate to evaluate community-level interventions for a variety of public health issues; this is particularly true for road traffic interventions, given that the dependencies in the observations over time and the non-stationarity of the data [29]. Given that there was a decrease in RT crash rate following the 2^nd^ intervention phase in the targeted cities but not at the national level, our study suggests that a focused multi-faceted community based road safety intervention program can be effective in reducing RT crashes in the areas in which interventions are targeted. However, given the national-level reduction in RT crashes that occurred after the 1^st^ intervention phase, it is possible that other factors outside of the specific interventions played a role in the changes in RT crashes over time. It will be important to determine if the decreases in RT crashes in these municipalities following the second phase of the intervention program were due in part to this project, and what other factors were involved. Although there has yet been no significant change in RT injury and mortality rates in either city following either phase of the intervention, there have only been 24 months of analysis following launching of the 2^nd^ phase and it is possible that effects will be seen in time. Another possible reason for the lack of change in RT injury and mortality rates despite the decrease in crash rates is that there has been a change only in the less-severe crashes causing property damage with minimal or no personal injuries; our study did not explore crashes by severity/type so future studies would need to explore this. In addition, further research is needed in assessing what aspects of this program were most influential in causing this change, and if a sustained reduction in RT crash rate will ultimately results in a decrease in RT mortality.
